# LncRNA *PELATON*, a Ferroptosis Suppressor and Prognositic Signature for GBM

**DOI:** 10.3389/fonc.2022.817737

**Published:** 2022-04-28

**Authors:** Haijuan Fu, Zhaoyu Zhang, Danyang Li, Qingqing Lv, Simin Chen, Zuping Zhang, Minghua Wu

**Affiliations:** ^1^ Hunan Cancer Hospital and the Affiliated Cancer Hospital of Xiangya School of Medicine, Central South University, Changsha, China; ^2^ The Key Laboratory of Carcinogenesis of the Chinese Ministry of Health, The Key Laboratory of Carcinogenesis and Cancer Invasion of the Chinese Ministry of Education, Cancer Research Institute, Central South University, Changsha, China; ^3^ Department of Clinical Laboratory, Yueyang Central Hospital, Yueyang, China; ^4^ Department of Pathogeny Biology, School of Basic Medical Science, Central South University, Changsha, China

**Keywords:** lncRNA, ferroptosis, *PELATON*, *LINC01272*, ROS

## Abstract

*PELATON* is a long noncoding RNA also known as long intergenic nonprotein coding RNA 1272 (*LINC01272*). The known reports showed that *PELATON* functions as an onco-lncRNA or a suppressor lncRNA by suppressing miRNA in colorectal cancer, gastric cancer and lung cancer. In this study, we first found that *PELATON*, as an onco-lncRNA, alleviates the ferroptosis driven by mutant *p53* and promotes mutant *p53*-mediated GBM proliferation. We also first confirmed that *PELATON* is a new ferroptosis suppressor lncRNA that functions as a ferroptosis inhibitor mainly by mutant *P53* mediating the ROS ferroptosis pathway, which inhibits the production of ROS, reduces the levels of divalent iron ions, promotes the expression of *SLC7A11*, and inhibits the expression of *ACSL4* and *COX2*.*PELATON* can inhibit the expression of *p53* in *p53* wild-type GBM cells and regulate the expression of *BACH1* and *CD44*, but it has no effect on *p53*, *BACH1* and *CD44* in *p53* mutant GBM cells. *PELATON* and *p53* can form a complex through the RNA binding protein EIF4A3. Knockdown of *PELATON* resulted in smaller mitochondria, increased mitochondrial membrane density, and enhanced sensitivity to ferroptosis inducers to inhibit GBM cell proliferation and invasion. In addition, we established a favourite prognostic model with *NCOA4* and *PELATON*. *PELATON* is a promising target for the prognosis and treatment of GBM.

## Introduction

Glioblastoma (GBM) is the most common malignant brain tumour of the central nervous system, accounting for approximately 45% of central nervous system tumours, with an annual incidence of 3.19 cases/100000 people (1–3). Recent studies have shown that the main factors affecting the prognosis of glioblastoma patients include the degree of surgical resection of the tumour tissue and the molecular classification of the tumour. With improvements in surgical accuracy and progress in tumour imaging, it is easier to distinguish glioblastoma from normal brain tissue and maximize the removal of tumour tissue. However, due to the invasive growth of glioblastoma, tumour cells often infiltrate normal brain tissue, resulting in treatment failure and recurrence (4). At present, the conventional treatment of glioblastoma patients mainly includes optimal and safe surgical resection of tumour tissue, followed by adjuvant radiotherapy and chemotherapy (5, 6). An increasing number of studies are exploring targeted and personalized therapies for glioblastoma, such as targeting DNA repair, tumour growth, apoptosis, invasion, and angiogenesis and overcoming resistance to chemotherapeutic drugs, including temozolomide (7–10). Despite this, recurrence and drug resistance of glioblastoma are still common, and recurrent tumour cells grow faster and more aggressively. In the past decade, the poor prognosis of patients with glioblastoma has not improved significantly, and the overall median survival time remains at 16-18 months (11). Therefore, according to the pathogenesis of glioblastoma, identifying new therapeutic targets and developing effective alternative clinical therapies are still urgent problems to be solved.

Ferroptosis was first proposed by Dr. Brent R. Stockwell in 2012 as an iron-dependent programmed cell death (PCD), which is different from autophagy, apoptosis, and necrosis (12–14). The process involves high levels of iron ions, accumulation of reactive oxygen species, changes in mitochondrial morphology and lipid peroxide metabolism genes (15–17). Ferroptosis is characterized by the depletion of glutathione and a decrease in glutathione peroxidase (GPX4) activity. As a result, lipid oxides cannot be metabolized by the GPX4-catalyzed glutathione reductase reaction, and bivalent iron ions oxidize lipids to produce reactive oxygen species (ROS) (13, 18, 19). Related studies have shown that, as a new mechanism of cell death, ferroptosis may be involved in the development of disorders such as cancer, neurodegenerative diseases, inflammatory diseases, cardiovascular diseases, and T cell immunity (14, 20, 21). One of the reasons for the high degree of malignancy and drug resistance of glioblastoma is that these tumours can effectively escape ferroptosis (22). The induction of glioblastoma ferroptosis molecules or the synthesis of small molecule drugs and nanomaterials provides new ideas for the treatment of glioblastoma (23–28). For example, loss of COPI coat complex subunit zeta 1 induces nuclear receptor coactivator 4 (NCOA4)-mediated autophagy and ferroptosis in glioblastoma cells (29). The curcumin analogues ALZ003 and quinkalim can lead to ferroptosis in glioma cells, thus opening new avenues for the treatment of temozolomide (TMZ)-resistant glioblastoma (30, 31). Iron oxide nanoparticles are safe and effective ferroptosis and apoptosis inducers and can be used as a combination therapy for glioblastoma (32, 33).

Long noncoding RNAs (lncRNAs) may promote or suppress the occurrence and development of tumours (34). They are involved in tumour invasion and metastasis, apoptosis, proliferation, drug resistance, and angiogenesis and regulate the expression of target genes at the transcriptional and posttranscriptional levels. An increasing number of studies have shown the important role of lncRNAs in the regulation of ferroptosis in cancer, but only a few have focused on GBM (23). At present, many reports have established the prognosis model of ferroptosis related genes in cancer including GBM by screening the differentially expressed ferroptosis related genes in the database and other bioinformatics analysis, so as to evaluate the tumour immune microenvironment and immune cell infiltration, which has good predictive value for the survival and immunotherapy of tumour patients (35, 36).Therefore, it is still urgent to further explore and study new molecules in GBM ferroptosis, so as to provide guidance for the clinical treatment of GBM.

In this study, we obtained 13 known ferroptosis mRNAs and 12 unreported ferroptosis lncRNAs, found that LncRNA *PELATON* and NCOA4 were prognostic ferroptosis genes, and constructed a favourite ferroptosis risk model for GBM. We also found that *PELATON* was mainly involved in the ROS ferroptosis pathway by mutant *p53*, and in *p53* mutant-type GBM cells, it suppressed the expression of ferroptosis driver genes and promoted the expression of ferroptosis suppressor genes. *PELATON* is a novel ferroptosis suppressor. Knockdown of *PELATON* promoted the production of ROS and the levels of divalent iron ions, the mitochondria decreased, the cell membrane density increased, and GBM cells displayed proliferation inhibition.

## Materials and Methods

### Collection of GBM Datasets

Based on The Cancer Genome Atlas (TCGA) database (https://cancergenome.nih.gov/), we performed transcriptome profiling by next-generation sequencing and obtained the corresponding clinical information of the GBM set. The GSE43378 (GPL570) dataset was obtained from the GEO database (https://www.ncbi.nlm.nih.gov/geo/), which contains gene expression and clinical data of GBM.

### WGCNA Analysis

A cluster dendrogram of the genes was constructed to check for outliers using the hclust function. After removing the outlier genes, the R package “weighted gene co-expression network analysis” (“WGCNA”) was used to establish the co-expression network of highly expressed genes (37). In our study, we used the pick Soft Threshold function to determine the soft-thresholding powers β over R2. Using the value of β for which the value of R2 is maximum with the transformed gene expression matrix, we constructed the adjacency matrix and topological overlap matrix (TOM). For the construction of the module, a dendrogram of genes was constructed with a dissTOM matrix using the hclust function with different colours. Based on the TOM dissimilarity measurements, we established an average hierarchical linkage clustering. Module dendrograms were built by setting the minimum genome to 30, and highly similar modules were merged by setting a cutoff of < 0.25. The dissimilarity of the module eigengenes was calculated using the module eigengenes function. The association between eigenvalues and FPI was assessed using Pearson’s correlation.

### Ferroptosis Potential Index (FPI)

The FPI was calculated according to the method of Liu Z et al. (38). We assessed the ferroptosis level, which was established based on the expression data for genes positively or negatively regulating ferroptosis. The enrichment score (ES) for a gene set that positively or negatively regulated ferroptosis was calculated using single-sample gene set enrichment analysis (ssGSEA) in the R package ‘GSVA’ (39), and the normalized differences between the ES of the positive components and negative components were defined as the FPI to computationally dissect the ferroptosis levels/trends in the tissue samples.

### GEPIA Analysis

Differentially expressed genes, OS, and FPS were integrated using Gene Expression Profiling Interactive Analysis 2 (GEPIA2, http://gepia2.cancer-pku.cn/) (40). We identified the differentially expressed genes with |log2FC| values > 1 and q values < 0.05 using LIMMA. OS and DFS were evaluated using the Kaplan–Meier method with the median cutoff and compared using the log-rank test.

### Enrichment Analysis

We utilized the “clusterprofiler” package to conduct Gene Set Enrichment Analysis (GSEA) analysis for GO enrichment and KEGG (41). KEGG pathway analysis was performed on ferroptosis genes using the R package “clusterprofiler” (41). Meanwhile, adjusted p<0.05 was regarded as statistically significant.

### Antibodies and Reagents

The reagents, chemicals, and antibodies used in this study were as follows: SLC7A11 (Abcam, ab175186, Massachusetts, US), COX2 (Abcam, ab179800, Massachusetts, US), GPX4 (Abcam, ab125066, US),ACSL4 (Abcam, ab155282, Massachusetts, US), BACH1 (Abcam, ab180853, Massachusetts, US), CD44 (Abcam, ab243894,Massachusetts,US), P53(Proteintech,CatNo.60283-2-Ig, China), GAPDH (Proteintech, 60004-1-Ig, Wuhan, China), DMSO (MP Biomedicals, 19605580, California, USA), and erastin (MedChemExpress, HY-15763, Shanghai, China).

### Tissue Collection, Glioblastoma Cell Lines and Primary Cell Culture

These procedures were performed as previously described in detail in our previous study (42, 43). Human clinical sample and data were collected from the Department of Neurosurgery, Central South University. All human experiments were performed in accordance with the Declaration of Helsinki and approved by the Joint Ethics Committee of the Central South University Health Authority. All subjects provided informed written consent. Primary tumour samples were minced about lmm3 with a GentleMACS Dissociator (Miltenyi Biotec). The cells were digested with trypsin and incubated at 37° for 10 minutes, then tissue suspension was filtered through the filter screen(Jet Biofil) to remove the undigested tissue residue, and centrifuged at 800 rpm for 5 ~ 8 minutes, Cells were cultured in DMEM/F12 containing 10% FBS, 5% CO2 and 37°C. Primary tumour cells were tested by GFAP, nestin, and CD133 staining and subcutaneous implantation in nude mice.

### Cell Transfection Assay

Cells with approximately 80% confluence were transiently transfected with 3.1- or 3.1-*PELATON* Plasmid, *PELATON*-siRNA ([226] 5′-GCAGCACAGUCACAUCCUATT-3′, [342] 5′-GCGCCUGUCCAGGACAAGUTT-3′, and [478] 5′-GCACAGAAGUCUCUUCCCUTT-3′). siRNAs were synthesized by RiboBio (Guangzhou, China). Cell transfection was performed using Lipofectamine 3000 (Invitrogen Life Technologies, Carlsbad, CA, USA) according to the manufacturer’s instructions.

### RT–qPCR

Total RNA was extracted from cells using TRI Reagent (Molecular Research Center, TR118, Cincinnati, OH 45212, USA), and its concentration and purity were determined using a Nanodrop2000 microultraviolet spectrophotometer. The extracted RNA was reverse transcribed into cDNA using the RevertAid RT Reverse Transcription Kit (Thermo Scientific, K1691, USA) according to the instructions of the manufacturer, and qPCR was carried out on a real-time fluorescence quantitative instrument (Bio–Rad, 788BR06968, USA). The gene-specific primers used are as follows:

*PELATON* Forward:5 ′ACAAAGATGAGACGCAGGCT 3′;
*PELATON* Reverse:5 ′GTTAAGGGCCCGGGAATCTG 3′;
*SLC7A11* Forward: 5′GGACAAGAAACCCAGGTGGT 3′;
*SLC7A11* Reverse: 5′GCAGATTGCCAAGATCTCAAGT 3′;
*COX2* Forward:5 ′CTATCCTGCCCGCCATCATC 3′;
*COX2* Reverse: 5 ′GGGATCGTTGACCTCGTCTG 3′;
*GPX4* Forward: 5′AGATCCAACCCAAGGGCAAG 3′;
*GPX4* Reverse: 5′GGAGAGACGGTGTCCAAACT 3′;
*ACSL4* Forward: 5′GCCCCTCCGATTGAAATCAC 3′;
*ACSL4* Reverse:5 ′AGCCGACAATAAAGTACGCAA 3′;
*BACH1* Forward: 5′ CGCCTCAGCTCTGGTTGAT 3′;
*BACH1* Reverse: 5′ ATCAGCCTGGCCTACGATTC 3′;
*CD44* Forward: 5′ AGTCACAGACCTGCCCAATG3′;
*CD44* Reverse: 5′ TTGCCTCTTGGTTGCTGTCT3′;
*GAPDH* Forward: 5 ′GAATGGGCAGCCGTTAGGAA 3′;
*GAPDH* Reverse: 5′AAAAGCATCACCCGGAGGAG 3′;

*GAPDH* was used as an internal control. The relative transcriptional levels of the target genes were calculated using the 2^−△△CT^ method. Datas were mean ± SEM for three independent experiments.

### Western Blot Analysis

Cells were lysed in RIPA buffer (Beyotime, Shanghai, China) for 30 min and centrifuged at 12,000 rpm for 10 min at 4°C, and the supernatants were collected. The protein concentration was determined using the BCA method (Thermo Scientific, 23222, USA). The proteins were separated by SDS–PAGE and transferred to a polyvinylidene fluoride membrane (Merck Millipore, ISEQ00010, USA). The PVDF membrane was incubated for 1 h in 5% skim milk powder at room temperature and then incubated with the corresponding anti-antibody overnight at 4°C. After washing thrice for 10 min with PBST, the membrane was incubated with the secondary antibody at 37°C for 1 h. The protein bands were visualized using enhanced chemiluminescence reagents (Abbkine, Wuhan, China, BMU102-CN). The ChemiDoc imaging system (Bio–Rad, USA) was used to capture the images and quantify the intensity of the protein fragments.

### Coimmunoprecipitation and RNA-Binding Protein Immunoprecipitation Assay

Cells were extracted with lysis buffer, and the supernatants were incubated with the indicated antibodies for 1 h at 4°C. Then, the samples were precipitated with agarose beads for 1 h at 4°C. The immunocomplexes were washed from agarose beads with Poly FLAG Peptide and then subjected to the second co-IP with the indicated antibodies and agarose beads. The final retrieved protein was detected by Western blotting. The coprecipitated RNAs were detected by RT–qPCR.

### Transmission Electron Microscopy

These procedures have been previously described in detail (42).

### Transwell Assay

The glioma cell suspension (1×10^6^ cell/ml, 100 μL) was added to the transwell chamber covered with Matrigel (Corning, 256234, USA), and 600 μl medium containing 15% FBS was added to the 24-well subplate chamber. The transwell chamber was removed after 48 h of culture and fixed with 4% formaldehyde for 30 min. The cells were stained with 0.1% crystal violet and washed thrice with PBS. Five microscope fields were photographed for each group, and the cell numbers were counted using ImageJ software. The experiment was repeated three times.

### Wound-Healing Assay

The glioma cells were inoculated into a 6-well plate and transfected for 48 h. A 2 mm width scratch was made in the middle of the tissue culture plate and cultured for another 48h. Photographs were taken at certain time points, and the scratch healing rate was calculated using ImageJ software. Datas were mean ± SEM for three independent experiments.

### Detection of Intracellular ROS Levels

To calculate the production of intracellular ROS, a reactive oxygen species detection kit (Biosharp, Shanghai, China) was used. First, the ROS probe H2DCFH-DA was diluted to 10 μM in serum-free culture medium, and 1 ml H2DCFH-DA working solution was added to each well at 37°C in the dark for 30 min. Then, the cells were washed with serum-free medium 3 times to fully remove H2DCFH-DA that did not enter the cells. Finally, the cells were observed under a fluorescence microscope and photographed.

### Iron Ion Detection

After protein extraction, the protein concentration was determined using the BCA method (Thermo Scientific, 23222, USA). Iron levels in the samples were determined using an iron ion detection kit (Leagene, Beijing, China) according to the manufacturer’s instructions. The corresponding reagents were added in turn and mixed gently at 37°C for 10 min, and the absorbance of the detection well was measured at 562 nm. Finally, the plasma and serum Fe (μM/L) were measured as follows: [Fe]= [A determination-(A serum blank × 0.970)]/A standard × 35.8.

### Fluorescence *In Situ* Hybridization (FISH)

Paraffin sections of glioma and normal brain tissues were baked at 42°C for 2 h, dewaxed with xylene, dehydrated in graded ethanol solutions (100%-95%-80%-50%-30%) for 5 min, treated with DEPC water for 2×5 min, and washed with PBS (pH 7.4) for 2 × 5 min. Afterwards, the sections were treated with 0.3% Triton X-100 for 15 min to permeabilize the membranes and washed with PBS for 2×5 min. Subsequently, the sections were digested with RNase-free protease K (20 g/ml) at 37°C for 20 min and washed with 100 mM Gly/PBS and PBS. Then, 4% paraformaldehyde (4°C) was added for 5 min to fix the samples. Triethanolamine buffer (100 mM, pH 8.0) containing 0.25% (w/v) acetic anhydride was discharged for 15 min and washed with PBS. Then, the following steps were performed using an *in situ* hybridization detection kit from RiboBio (Guangzhou, China) according to the instructions of the manufacturer to avoid light in the whole process, and glioma cells were used as in the RiboBio FISH kit. The sections were analysed using a confocal microscope.

### Statistical Analysis

The most significant ferroptosis gene signatures associated with the OS of patients with GBM were identified using the Lasso-penalized Cox regression model (44). We set 10-fold cross-validation as the criterion to prevent overfitting with the penalty parameter lambda. Then, we used the time-dependent receiver operating characteristic (ROC) curve and the area under the curve (AUC) to identify the prognostic accuracy of the two-gene signature model in the discovery set and internal set with the package “survival ROC” (45). To separate patients into high-risk and low-risk score groups, we set the median risk score as the cutoff value and then used Kaplan–Meier survival analysis and the log-rank test to evaluate differences in OS between the two groups. The nomogram was established based on the “regplot” package.

SPSS 21.0 (IBM Corp., Armonk, NY, USA) and Prism 7.0 were used for statistical analysis. Statistical analysis was performed using the t test and analysis of variance. Statistical significance was set at p < 0.05. The measured data were expressed as “mean ± SEM”. A single factor analysis of variance (ANOVA) was used for comparison among the groups. Datas were mean ± SEM for three independent experiments.

## Results

### Identification of Ferroptosis LncRNAs in GBM

**Figure 1:**
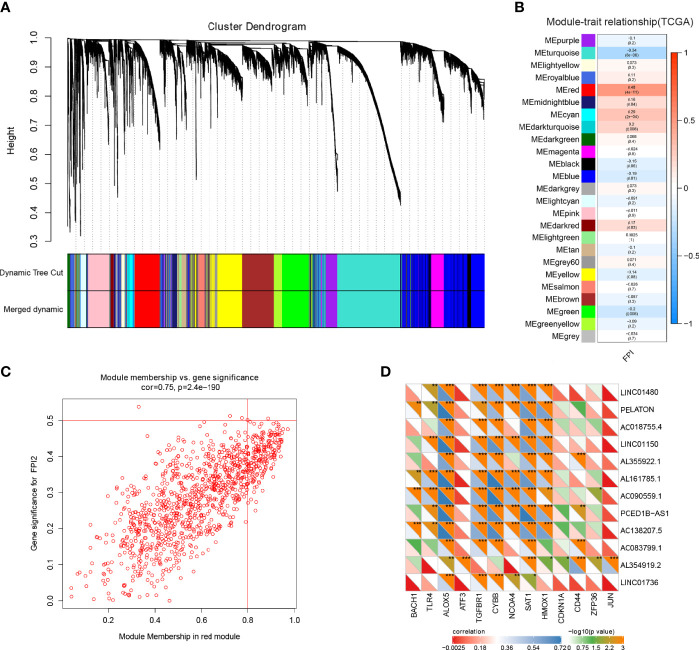
Ferroptosis gene identification in GBM. **(A)** Dendrogram of all highly expressed genes clustered based on a dissimilarity measure (1-TOM) together with assigned module colours in the GBM cohort of TCGA. **(B)** Heatmap of the correlation between module genes and FPI in GBM. Each range contains the Pearson correlation coefficient and P value. **(C)** Significant correlation between the module membership of the red module and FPI gene significance. **(D)** Identification and correlation of 13 known ferroptosis-associated mRNAs and 12 unreported ferroptosis lncRNAs by Pearson correlation analysis from 610 DEGs (*p < 0.05; **p < 0.001; ***p < 0.001).

To identify the ferroptosis genes of GBM, WGCNA analysis was used to identify the key module correlated with ferroptosis in GBM. Based on mRNAs found to be expressed at high levels (average expression of FPKM >0.5) in the TCGA cohort, which includes 18293 genes, 25 co-expression modules were constructed (**Supplementary Figures 1A-D**) (37), in which the red module containing 1049 genes showed the highest correlation with ferroptosis (**Figures 1A, B**). There was a highly significant correlation between the module membership (MM) of the red module and FPI gene significance (GS) (**Figure 1C**) (38). In addition, we used a two-sided hypergeometric test to find 12 ferroptosis driver sets (FDR=7.32eE-06) and 8 suppressor sets (FDR=0.000345) in the red module (**Supplementary Figures 1E, F**) (46). Furthermore, the differentially expressed gene (DEG) analysis indicated that 58% (610/1049) of the ferroptosis genes of the red module were differentially expressed in GBM (**Supplementary Figure 1G**). We obtained 13 known ferroptosis mRNAs and 12 unreported ferroptosis lncRNAs from 610 DEGs (**Figure 1 D** and **Table 1**).

**Table 1:** Screening of 12 LncRNAs and 13 mRNAs associated with Ferroptosis.

RNA	Gene abbreviation	Full name	NCBI Entrez Gene	Ensembl
LncRNA	LINC01480	Long Intergenic Non-Protein Coding RNA 1480	101927931	ENSG00000270164
	PELATON	Plaque Enriched LncRNA In Atherosclerotic And Inflammatory Bowel Macrophage Regulation	100506115	ENSG00000224397
	AC018755.4	NA	NA	ENSG00000273837
	LINC01150	Long Intergenic Non-Protein Coding RNA 1150	101927624	ENSG00000229671
	AL355922.1	NA	NA	ENSG00000136315
	AL161785.1	NA	NA	ENSG00000224307
	AC090559.1	NA	NA	ENSG00000255197
	PCED1B-AS1	PCED1B Antisense RNA 1	100233209	ENSG00000247774
	AC138207.5	NA	NA	ENSG00000265743
	AC083799.1	NA	NA	ENSG00000203644
	AL354919.2	NA	NA	ENSG00000254545
	LINC01736	Long Intergenic Non-Protein Coding RNA 1736	101927532	ENSG00000228058
mRNA	BACH1	BTB Domain And CNC Homolog 1	571	ENSG00000156273
	TLR4	Toll Like Receptor 4	7099	ENSG00000136869
	ALOX5	Arachidonate 5-Lipoxygenase	240	ENSG00000012779
	ATF3	Activating Transcription Factor 3	467	ENSG00000162772
	TGFBR1	Transforming Growth Factor Beta Receptor 1	7046	ENSG00000106799
	CYBB	Cytochrome B-245 Beta Chain	1536	ENSG00000165168
	NCOA4	Nuclear Receptor Coactivator 4	8031	ENSG00000266412
	SAT1	Spermidine/Spermine N1-Acetyltransferase 1	6303	ENSG00000130066
	HMOX1	Heme Oxygenase 1	3162	ENSG00000100292
	CDKN1A	Cyclin Dependent Kinase Inhibitor 1A	1026	ENSG00000124762
	CD44	CD44 Molecule (Indian Blood Group)	960	ENSG00000026508
	ZFP36	ZFP36 Ring Finger Protein	738	ENSG00000128016
	JUN	Jun Proto-Oncogene, AP-1 Transcription Factor Subunit	3725	ENSG00000177606

### The Favourite Ferroptosis Risk Model for GBM

To determine whether the above 25 ferroptosis genes are associated with the clinical prognosis of patients with GBM, we used survival coxph function to perform univariable Cox proportional hazard regression on the TCGA cohort. Then, by a single factor test followed by Lasso regression analysis, two prognostic ferroptosis genes were identified: LncRNA *PELATON* and *NCOA4* (**Supplementary Figures 2A–C**). Combining the regression coefficients with gene expression values, a risk score formula was created as follows: risk score = -0.69641*NCOA4+ 0.35167*PELATON.

**Figure 2:**
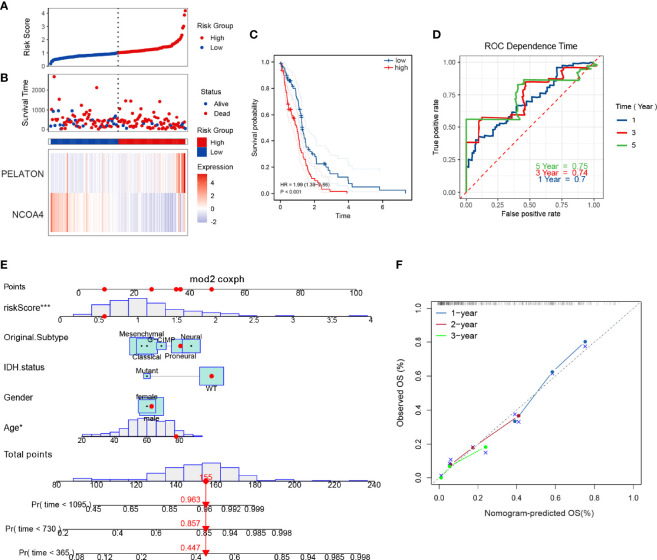
Ferroptosis risk prognosis model with *NCOA4* and *PELATON.* The distribution of risk factors **(A, B)**, Kaplan–Meier survival analysis **(C)**, and time-dependent ROC curves at 1, 3, and 5 years **(D)** between patients at high and low risk based on the *NCOA4* and *PELATON* prognostic models in the TCGA discovery set. **(E)** Nomogram integrating the ferroptosis risk score, age, sex, original subtype and IDH status. **(F)** Calibration curve for predicting OS at 1, 2 and 3 years.

To evaluate the predictive ability of the ferroptosis risk model with *NCOA4* and LncRNA *PELATON* for patients with GBM, we performed Kaplan–Meier survival and time-dependent ROC analysis in the discovery set of TCGA (n=161) and the internal set of GSE43378 (n=50). In the discovery set, the higher the risk score (**Figure 2A**), the greater the number of deaths (**Figure 2B**), and the lower the survival rate of patients with GBM (**Figure 2C**), the predictive accuracy of the signature was 0.70, 0.74 and 0.75 at 1, 3, and 5 years, respectively (**Figure 2D**). We obtained consistent results in the internal set (**Supplementary Figures 2D–G**).

To develop a clinically applicable tool that can easily assess the prognosis of patients with GBM, we established a graphical nomogram. The nomogram was based on the discovery set for predicting overall survival (OS). The independent prognostic factors were age, sex, original subtype, isocitrate dehydrogenase (IDH) status, and ferroptosis risk score. A nomogram capable of predicting the OS probabilities of GBM at 1, 2 and 3 years was constructed (**Figure 2E**). The calibration curves at 1, 2 and 3 years showed good consistency between actual observation and prediction by the nomogram (**Figure 2F**).

### *PELATON* in the ROS-Mediated Ferroptosis Pathway by Mutant *p53*

**Figure 3:**
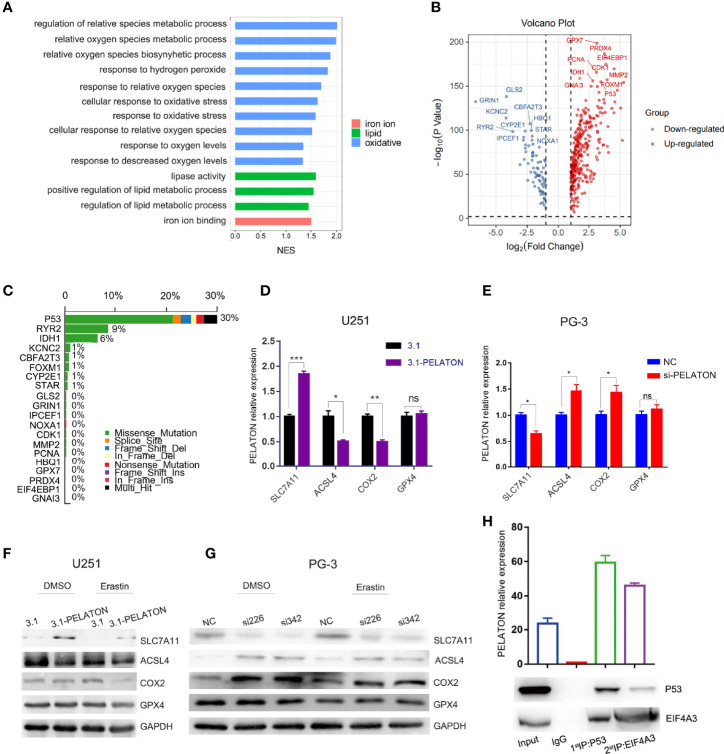
*PELATON* in the ROS-mediated ferroptosis pathway by mutant *p53*. **(A)** GO analysis of biological processes related to *PELATON* in GBM. **(B)** Top 20 genes involved in ROS biosynthesis that are differentially expressed between GBM and normal brain tissue. **(C)** Gene mutation or overexpression in human GBM. RT-qPCR analysis of *ALSL4*, *COX2*, *SLC7A11* and *GPX4* when *PELATON* was overexpressed **(D)** or knocked down **(E)** in glioblastoma cells (*p< 0.05, **p < 0.01; ***p < 0.001; ns, not significant). Datas were mean ± SEM for three independent experiments. western blot analysis of ALSL4, *COX2*, *SLC7A11* and *GPX4* when *PELATON* was overexpressed **(F)** or knocked down **(G)** in glioblastoma cells. independent experiment was repeated for three times. **(H)**. The *PELATON*-*EIF4A3*-*P53* complex was detected by two-step immunoprecipitation and RT–PCR, Datas were mean ± SEM for three independent experiments.

To reveal the effects of *PELATON* on GBM progression, we performed Gene Ontology (GO) and KEGG analyses by Gene Set Enrichment Analysis (GSEA) on RNA-seq data from the TCGA cohort in GBM. Both GO and KEGG analyses suggested that reactive oxygen species (ROS) biosynthesis was mainly in response to the ferroptosis pathway involved by *PELATON* (**Figure 3A** and **Supplementary Figures 3A–C**). To identify which is likely the most important molecule of ROS biosynthesis involved in the ferroptosis suppressor *PELATON*, we analysed the top 20 genes that are differentially expressed between GBM and normal brain tissue (**Figure 3B**) and then wanted to determine which of these is most commonly mutated or overexpressed in human GBM, which revealed *P53*, *RYR2* and *IDH1* at the top of this analysis, with a mutation rate of *p53* up to 30% (**Figure 3C**).

Since wild-type *p53* is a tumour suppressor gene that mainly acts as a transcription factor and prevents oncogenesis, its coding gene *p53* is highly mutated, and its activity is almost abrogated in ~50% of human cancers (47). Combining **Figure 5C**, next, we mainly focused on the *p53* mutant-type GBM cells to explore the function of *PELATON*. By hTFtarget database analysis, we determined that *P53* regulates ferroptosis-related target genes, such as the ferroptosis suppressor genes *SLC7A11*, *GPX4*, and *CD44* and the ferroptosis driver genes *ACSL4* and *BACH1*. Then, pcDNA3.1-*PELATON* was transfected into GBM U251 cells, which is a *p53* mutant-type GBM cell line with lower levels of *PELATON* expression (**Supplementary Figure 3D**), and *PELATON* was knocked down in PG-3 cells, which are primary cultured *p53* mutant-type GBM cells with high levels of *PELATON* expression (**Supplementary Figure 3E**). We found that the overexpression of *PELATON* inhibited *ACSL4* expression and promoted *SLC7A11* expression in U251 cells (**Figures 3D, F**), whereas knockdown of *PELATON* promoted *ACSL4* expression and inhibited *SLC7A11* expression in PG-3 cells (**Figures 3E, G**). In wild-type *p53* primary cultured GBM PG-1 cells, *PELATON* inhibited the expression of *BACH1* and *CD44* (**Supplementary Figures 3F, H**), but *PELATON* had no effect on the expression of *GPX4*, *BACH1* and *CD44* in the *p53* mutant GBM cells, such as PG-3 and PG-2 (**Figures 3D–G** and **Supplementary Figures 3G, I**), suggesting that the mutant site of *P53* may affect the binding of *P53* and target genes. In addition, in mutant *p53* GBM cells, we also found that the overexpression of *PELATON* inhibited the expression of the ferroptosis-driven gene *COX2* (**Figures 3D, F**), and knockdown of *PELATON* promoted the expression of *COX2* (**Figures 3E, G**).

Bioinformatics correlation analysis showed that *PELATON* was negatively correlated with *p53* (**Supplementary Figure 4A**), and wild-type or mutant *P53* in GBM patients did not affect *PELATON* expression (**Supplementary Figure 4B**). Further results showed that *PELATON* inhibited the expression of wild-type *p53* in GBM PG-1 cells but had no effect on mutant *p53* in GBM PG-2 and PG-3 cells (**Supplementary Figures 3H, I**). In wild-type *p53* GBM PG-1 cells, simultaneous overexpression of *PELATON* and *P53* inhibited *PELATON*’s regulation of *BACH1* and *CD44* (**Supplementary Figure 4C**). Further research and bioinformatics prediction found that *PELATON* and *P53* can form a complex through the RNA-binding protein EIF4A3, which suggests a possible mechanism by which *PELATON* mediates ferroptosis in *p53* wild-type or mutant GBM cells (**Figure 3H** and **Supplementary Tables 2**, **3**) (48). The above data suggested that *PELATON* suppressed the expression of ferroptosis driver genes and promoted the expression of ferroptosis suppressor genes, suggesting that *PELATON* may be a ferroptosis suppressor.

### *PELATON* Is a Novel Ferroptosis Suppressor in GBM

**Figure 4:**
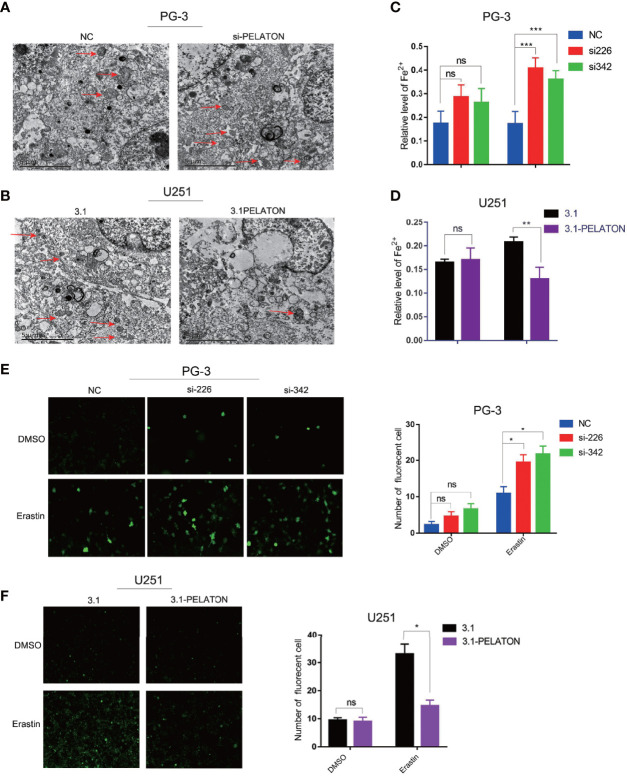
*PELATON* is a novel ferroptosis suppressor in GBM. The effect of downregulation **(A)** or upregulation **(B)** of *PELATON* on the morphology of mitochondria in glioblastoma cells assayed by transmission electron microscopy, independent experiment was repeated for three times. The effect of downregulation **(C)** or upregulation **(D)** of *PELATON* on the levels of iron in glioblastoma cells (**p < 0.01; ***p < 0.001; ns: not significant), Datas were mean ± SEM for three independent experiments. The effect of downregulation **(E)** or upregulation **(F)** of *PELATON* on the levels of reactive oxygen species in glioblastoma cells (*p < 0.05; ns, not significant), Datas were mean ± SEM for three independent experiments. Fluorescence intensity of the active oxygen probe photographed by laser confocal microscopy (left) and quantification of the fluorescence intensity of the reactive oxygen species probe (right).

Transmission electron microscopy observation showed that mitochondria decreased, the cell membrane density increased, and cristae decreased or even disappeared after *PELATON* was knocked down in PG-3 primary GBM cells, whereas pcDNA3.1-*PELATON* U251 cells had a relatively normal mitochondrial morphology (**Figures 4A, B**). The increase in reactive oxygen species and divalent iron ions is a sign of ferroptosis. By determining the levels of ROS and divalent iron ions, we found that knockdown of *PELATON* PG-3 in primary GBM cells promoted the production of ROS and induced the levels of divalent iron ions (**Figures 4C, E**), even after treatment of GBM cells with the ferroptosis inducer erastin (10 μM) for 4 h, and the opposite effect was observed in pcDNA3.1-*PELATON* U251 cells (**Figures 4D, F**).

### *PELATON* Promotes GBM Cell Phenotypes

**Figure 5:**
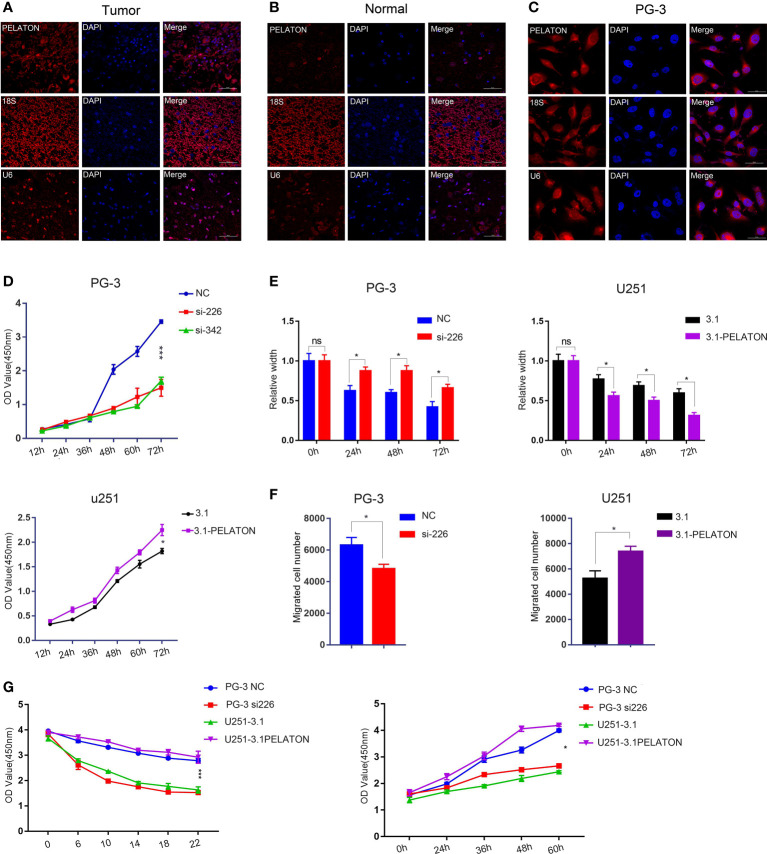
*PELATON* promotes GBM cell phenotypes. Fluorescence *in situ* hybridization of *PELATON* in GBM tissues **(A)**, normal brain tissues **(B)**, and GBM cells **(C)**. **(D)** CCK8 analysis of *PELATON* knockdown or overexpression on glioblastoma cell proliferation (*p < 0.05; ***p < 0.001), independent experiment was repeated for three times. **(E)** Quantification of the migration ability of PG-3 (left) and U251 (right) cells after interference or overexpression with *PELATON* (*p < 0.05; ns, not significant), Datas were mean ± SEM for three independent experiments. **(F)** Quantification of the number of invasive cells after knockdown (left) or overexpression (right) of *PELATON* in glioblastoma cells (*p< 0.05), Datas were mean ± SEM for three independent experiments. **(G)** CCK8 analysis of *PELATON* overexpression or knockdown on proliferation and sensitivity to the ferroptosis inducer erastin (*p< 0.05, ***p < 0.001), Datas were mean ± SEM for three independent experiments.

Although there is a known relationship between *NCOA4* and ferroptosis (29, 49, 50), there is no information about *PELATON* in ferroptosis. *PELATON* is a long intergenic nonprotein coding RNA1272 (also known as *LINC01272*). Few studies have indicated that it promotes cancer cell migration and invasion, such as gastric cancer (51, 52), colorectal cancer (53), and non-small-cell lung cancer (54), but there is no report in GBM. *PELATON* showed significantly higher expression in GBM tissues and primary GBM cells, which were named PG-2, PG-3, PA-2, and PA-3 (42), and was mainly located in the plasma membrane of GBM cells (**Supplementary Figure 5A** and **Figures 5A–C**). Patients with the highest 20% *PELATON* expression had significantly shorter overall survival and free-progression survival (FPS) than the remaining GBM patients (**Supplementary Figures 5B, C**).

The CCK8 assay showed that pcDNA3.1-*PELATON* increased U251 cell proliferation, comparable with that of pcDNA3.1 U251 cells, which have relatively low expression *PELATON*. Knockdown of *PELATON* inhibited the proliferation of PG-3 primary GBM cells, which have relatively high *PELATON* expression (**Figure 5D**). Wound-healing and transwell assays showed that pcDNA3.1-*PELATON* promoted active migration and invasion in U251 cells and vice versa (**Figures 5E, F** and **Supplementary Figures 5D, E**). We also assessed the effect of *PELATON* on GBM cell proliferation in the presence of the ferroptosis inducer erastin, in which ROS- and iron-dependent signalling is required for erastin-induced ferroptosis. pcDNA3.1-*PELATON* U251 cells and PG-3 primary GBM cells with high *PELATON* expression resisted ferroptosis induced by erastin in a concentration- and time-dependent manner, whereas knockdown or low *PELATON* expression promoted ferroptosis induced by erastin to inhibit PG-3 cell proliferation (**Figure 5G**).

## Discussion

*PELATON* is a long noncoding RNA also known as long intergenic nonprotein coding RNA 1272 (*LINC01272*), small integral membrane protein 25 (*SMIM25*), or GC-related lncRNA 1 (*GCRL1*). A handful of reports indicated that *PELATON* has dual functions as an oncogene or a suppressor gene by acting as a miRNA sponge (53, 55, 56). *PELATON* promotes metastasis of colorectal cancer or gastric cancer by targeting the *miR-876*/ITGB2 axis (53) or *miR-885-*3p/CDK4 *(* 52). *PELATON* also inhibits lung cancer and non-small cell lung cancer by targeting the *miR-7-5p*/CRLS1 axis or by inhibiting *miR-1303 (* 52, 57). Our research first showed that *PELATON* is highly expressed in gliomas and functions as an oncogene to promote the proliferation and invasion of *P53* mutant-type GBM cells by inhibiting ferroptosis.

Ferroptosis is an iron-dependent PCD in which cells die because of the toxic accumulation of lipid ROS (58). In cancer, the goal of treatment is to activate ferroptosis and cause the death of tumour cells that are resistant to other PCDs. An increasing number of studies have identified several drivers and suppressors of ferroptosis. Zhou et al. annotated the genes in 784 articles on the ferroptosis FerrDb website and found 253 regulatory factors, including 108 drivers, 69 suppressors, 35 inducers, and 41 inhibitors (46). It is expected that interfering with ferroptosis-related drivers and suppressors, inducers and inhibitors will provide new approaches for the treatment of cancer and metabolic diseases (23–25, 30). The common ferroptosis drivers are *PTGS2*/*COX2 (* 59), *ACSL4 (* 1), *NCOA4*, *BECN1 (* 60), *BACH1* and *P53* (58, 61). *P53* promotes ferroptosis by inhibiting the expression of *SLC7A11* or increasing the expression of SAT1, GLS2, and PTGS2. *P53* also inhibits ferroptosis by directly inhibiting the activity of dipeptidylpeptidase-4 or by inducing the expression of cyclin-dependent kinase inhibitor 1A (61). Ferroptosis suppressors have also achieved good research results, such as nuclear factor, erythroid 2-like 2 (*NRF2*) (20, 62–66), *SLC7A11 (* 15), *CD44* and *GPX4 (* 13, 18, 58, 61). The cystine/glutamate antiporter *SLC7A11* (also known as *xCT*) is used to uptake cysteine for glutathione biosynthesis and antioxidant defence. *SLC7A11* is a ferroptosis suppressor gene that is overexpressed in many human cancers (16). Drugs that target *SLC7A11* and block cystine uptake can cause ferroptosis. *SLC7A11* is regulated by the transcription factors NRF2, ATF4, and *P53 (* 61). *GPX4*, a type of glutathione peroxidase (GPX), is a key inhibitor of ferroptosis. Overexpression of *GPX4* endows tumour cells with resistance to ROS-induced cell death, while silencing *GPX4* sensitizes tumour cells (16, 64, 67). In our study, we first confirmed that *PELATON* is a novel ferroptosis suppressor that functions as a ferroptosis inhibitor mainly by mutant *p53* mediating the ROS ferroptosis pathway. In *p53* mutant-type GBM cells, *PELATON* inhibits the production of ROS, reduces the levels of divalent iron ions, promotes the expression of *SLC7A11*, and inhibits the expression of *ACSL4* and *COX2*. GBM cells with *PELATON* knockdown showed smaller mitochondria, increased mitochondrial membrane density, and decreased mitochondrial cristae. To explore the possible mechanism between *PELATON* and P53, we found that *PELATON* and *P53* can form a complex through the RNA binding protein EIF4A3 (*PELATON*- EIF4A3- P53). *EIF4A3* is reported to be a new anticancer target whose consumption or inhibition will activate *p53* and inhibit the growth of cancer cells. *PELATON* may inhibit the RNA and protein expression of P53 through the *PELATON*-EIF4A3-P53 complex to inhibit GBM ferroptosis, which suggests a possible mechanism by which *PELATON* mediates ferroptosis in *p53* wild-type or mutant GBM cells.

It is well known that the resistance of cancer cells to chemotherapy is a major obstacle in cancer treatment. Activation of the ferroptosis pathway can induce cancer cell death, especially in the case of drug resistance, and enhance the sensitivity of tumours to chemotherapeutic drugs (68). Studies have shown that TMZ combined with erastin can significantly improve antitumor activity, which reflects the importance of ferroptosis in the treatment of gliomas (31, 69, 70). Our experiments confirmed that knockdown of *PELATON* enhanced the sensitivity of GBM cells to erastin and inhibited the proliferation of tumour cells. Overexpression of *PELATON* inhibited the effect of erastin on glioma cells. It is suggested that interference with *PELATON* may provide a new target for treating glioma patients.

Nowadays, many reports screened differentially expressed genes of ferroptosis in the database, and then conduct enrichment analysis, interactive network analysis, univariate and multivariate Cox regression analysis to establish the prognosis model to predict the overall survival time, tumour immune microenvironment and immune cell infiltration (71–73). However, the study of ferroptosis in GBM needs to be further deepened. We not only screened 12 lncRNAs which closely related to ferroptosis, but also proposed a ferroptosis prognostic model with *NCOA4* and *PELATON* for patients with GBM, risk score = -0.69641*NCOA4+ 0.35167*PELATON. The higher the risk score is, the greater the death rate among patients with GBM. The survival rate of patients with GBM in the high-risk group was significantly lower than that in the low-risk group. Compared with other methods that require multiple genes for risk scoring to determine the survival of patients (74), we only use two genes to predict the effect, which is relatively accurate, predictive accuracy of the signature was 0.70, 0.74 and 0.75 at 1, 3, and 5 years.

In conclusion, we confirmed that *PELATON* is a new ferroptosis suppressor and an oncogene and established a prognostic model and diagram of ferroptosis in GBM patients with *NCOA4* and *PELATON*, provided that *PELATON* alleviates ferroptosis driven by wild-type or mutant *p53* and suppresses wild-type or mutant *p53*-mediated GBM proliferation. Knockdown of *PELATON* enhances the sensitivity to ferroptosis inducers to inhibit GBM cell proliferation and invasion. *PELATON* is an important target for the prognosis and treatment of GBM.

## Data Availability Statement

Publicly available datasets were analyzed in this study. This data can be found here: https://cancergenome.nih.gov/, https://www.ncbi.nlm.nih.gov/geo/ (GSE43378).

## Ethics Statement

We obtained GBM patients tumour samples and clinical data from Xiangya Hospital, Central South University, and all patients provided informed permission for the collection and use of these samples.

## Author Contributions

HJF mainly performed the experiments, and ZYZ mainly performed the information analysis. DYL, QQL, SMC and ZPZ helped with the experiments, and MHW proofread the manuscript. All authors contributed to the article and approved the submitted version.

## Funding

We thank the support from the National Natural Science Foundation of China (82073096), Key Research and Development Plan of Hunan Province (2020SK2053), Graduate Research and Innovation Projects of Hunan Province (1053320182361), and the Fundamental Research Funds for the Central Universities of Central South University(2021zzts0932).

## Conflict of Interest

The authors declare that the research was conducted in the absence of any commercial or financial relationships that could be construed as a potential conflict of interest.

## Publisher’s Note

All claims expressed in this article are solely those of the authors and do not necessarily represent those of their affiliated organizations, or those of the publisher, the editors and the reviewers. Any product that may be evaluated in this article, or claim that may be made by its manufacturer, is not guaranteed or endorsed by the publisher.

## Supplementary Material

Supplementary Figure 1 Ferroptosis-related gene clustering and identification in GBM. Sample gene clustering to detect seven outliers **(A)**. The FPI of gene clustering was calculated **(B)**. Set the soft-thresholding value to 4 (scale free R2 = 0.9, mean connectivity=115.71) **(C)** and cut height to 0.25 **(D)**. Intersection of ferroptosis driver and suppressor gene sets in FerrDb. The enrichment results of the constructing module in ferroptosis driver **(E)** and suppressor **(F)** gene sets (FDR<0.05). **(F)** Intersecting genes between DEGs (left, 6093) of GBM and the red module (right, 1050); 610 overlapping genes were selected (| Log2 (fold change) | ≥ 1, and p < 0.05).

Supplementary Figure 2 Ferroptosis-related gene identification of *NCOA4* and *PELATON.* **(A)** Results of the univariate Cox regression analyses of OS in the TCGA cohort. Seven genes (*PELATON, NCOA4, AL354919.2, HMOX1, AL355922.1, CD44, and ALOX5*) were identified. **(B)** LASSO coefficient plot of 25 genes (13 mRNAs and 12 lncRNAs) correlated with ferroptosis. **(C)** The optimal parameter (λ) was chosen by cross validation. The distribution of risk factors **(D–E)**., Kaplan–Meier survival analysis **(F)**, and time-dependent ROC curves at 1, 3, and 5 years **(G)** between patients at high and low risk based on the *NCOA4* and *PELATON* prognostic models in the internal set GSE43378.

Supplementary Figure 3
*PELATON* regulates *BACH1* and *CD44* in *p53*-mediated ferroptosis. **(A–C)** KEGG analysis of the ferroptosis signalling pathway related to *PELATON* in GBM. **(D)** The expression of *PELATON* in U251 cells after treatment with 3.1 or 3.1-*PELATON* (***p < 0.001), Datas were mean ± SEM for three independent experiments. **(E)** The expression of *PELATON* in PG-3 glioma primary cells after treatment with siRNAs (si226, si342, and si478), Datas were mean ± SEM for three independent experiments. (**p < 0.01). **(F–G)** RNA level changes of *BACH1* and *CD44* when *PELATON* was knocked down or overexpressed in glioblastoma cells, Datas were mean ± SEM for three independent experiments. (*p < 0.05, ***p < 0.001, ns: not significant). **(H–I)** Protein level changes of *BACH1*, *CD44* and *P53* when *PELATON* was knocked down or overexpressed in glioblastoma cells, independent experiment was repeated for three times.

Supplementary Figure 4 Regulatory Relationship between *PELATON* and *P53*. **(A)** Bioinformatics correlation between *PELATON* and *P53*. **(B)** The difference in *PELATON* expression between wild-type and mutant *P53* in GBM patients. **(C)** western blot analysis of *BACH1*, *CD44* and *P53* when *PELATON* and *P53* were overexpressed in glioblastoma cells, independent experiment was repeated for three times.

Supplementary Figure 5 Association of *PELATON* expression between patient prognosis and GBM cell phenotype. **(A)** RT-qPCR analysis of *PELATON* in glioma primary cells (PG-1, PG-2, PG-3, PA-1, PA-2, PA-3) and glioma cell lines (U118, U251). PG-1, PG-2, and PG-3 are primary cells from patients with glioblastoma, and PA-1, PA-2, and PA-3 are primary cells from patients with astrocytoma. Datas were mean ± SEM for three independent experiments. Kaplan–Meier curves showing overall survival **(B)** and disease-free survival **(C)** of patients with GBM stratified based on *PELATON* expression levels (p <0.05 p.adjust<0.25). **(D)** The effect of *PELATON* overexpression or knockdown on the invasion ability of glioblastoma cells (left), independent experiment was repeated for three times. **(E)** Migration ability of PG-3 and U251 cells after interference or overexpression with *PELATON*. Photos were taken at 0, 24, 48 and 72 hours, independent experiment was repeated for three times.

## Abbreviations

FPI: ferroptosis potential index; GBM: Glioblastoma; PCD: programmed cell death; GPX4: glutathione peroxidase; ROS: reactive oxygen species; NCOA4: Nuclear Receptor Coactivator 4; TMZ: temozolomide; lncRNAs: Long non-coding RNAs; ACSL4: Acyl-CoA Synthetase Long Chain Family Member 4; COX2: Prostaglandin-Endoperoxide Synthase 2; SLC7A11: Solute Carrier Family 7 Member 11; MM: module membership; GS: gene significance; KEGG: Kyoto Encyclopedia of Genes and Genomes; OS: overall survival; IDH: Isocitrate Dehydrogenase; FPS: free-progression survival; GO: Gene Ontology; GSEA: Gene Set Enrichment Analysis; TCGA: The Cancer Genome Atlas; ES: enrichment score; ssGSEA: single-sample gene set enrichment analysis; TOM: topological overlap matrix.
