# Supplementary figures and images for: LncRNA PELATON, a Ferroptosis Suppressor and Prognositic Signature for GBM
Source: Front Oncol. 2022 Apr 28;12:817737. doi: 10.3389/fonc.2022.817737 (PMC9097896; doi:10.3389/fonc.2022.817737)

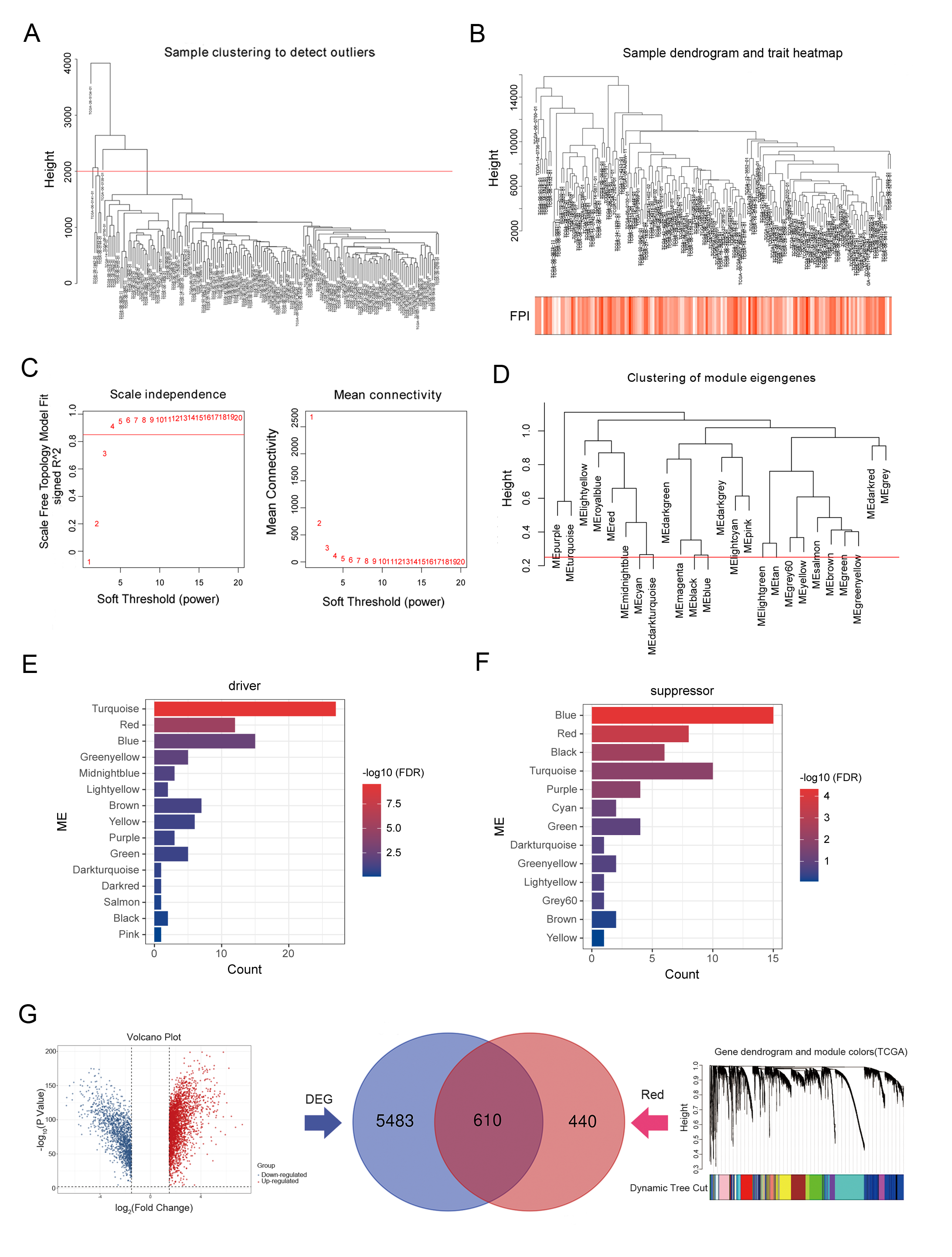

Supplement: Supplementary Figure 1 — Ferroptosis-related gene clustering and identification in GBM. Sample gene clustering to detect seven outliers (A). The FPI of gene clustering was calculated (B). Set the soft-thresholding value to 4 (scale free R2 = 0.9, mean connectivity=115.71) (C) and cut height to 0.25 (D). Intersection of ferroptosis driver and suppressor gene sets in FerrDb. The enrichment results of the constructing module in ferroptosis driver (E) and suppressor (F) gene sets (FDR<0.05). (F) Intersecting genes between DEGs (left, 6093) of GBM and the red module (right, 1050); 610 overlapping genes were selected (| Log2 (fold change) | ≥ 1, and p < 0.05). [file Image_1.tif]

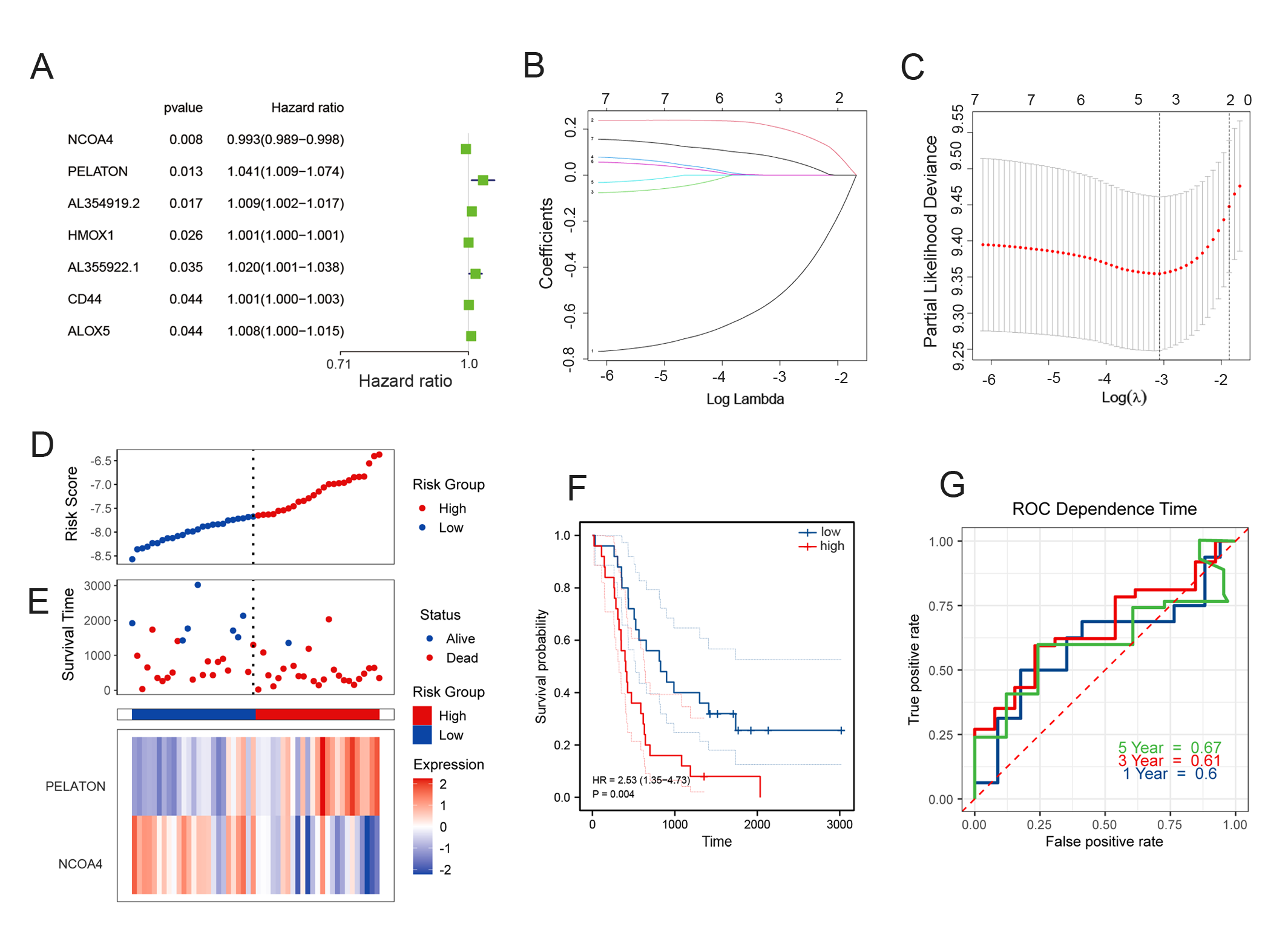

Supplement: Supplementary Figure 2 — Ferroptosis-related gene identification of NCOA4 and PELATON. (A) Results of the univariate Cox regression analyses of OS in the TCGA cohort. Seven genes (PELATON, NCOA4, AL354919.2, HMOX1, AL355922.1, CD44, and ALOX5) were identified. (B) LASSO coefficient plot of 25 genes (13 mRNAs and 12 lncRNAs) correlated with ferroptosis. (C) The optimal parameter (λ) was chosen by cross validation. The distribution of risk factors (D–E)., Kaplan–Meier survival analysis (F), and time-dependent ROC curves at 1, 3, and 5 years (G) between patients at high and low risk based on the NCOA4 and PELATON prognostic models in the internal set GSE43378. [file Image_2.tif]

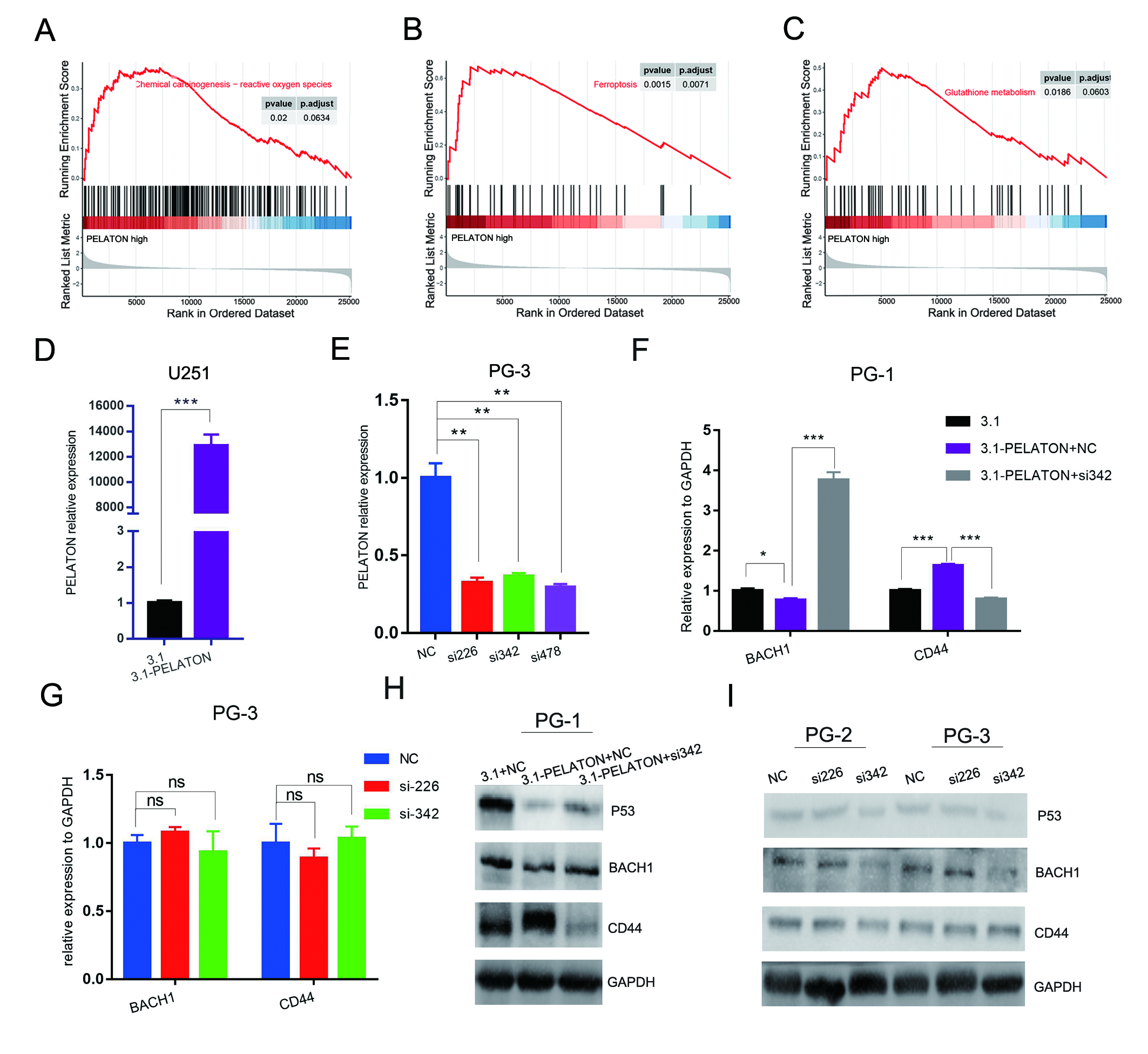

Supplement: Supplementary Figure 3 — PELATON regulates BACH1 and CD44 in p53-mediated ferroptosis. (A–C) KEGG analysis of the ferroptosis signalling pathway related to PELATON in GBM. (D) The expression of PELATON in U251 cells after treatment with 3.1 or 3.1-PELATON (***p < 0.001), Datas were mean ± SEM for three independent experiments. (E) The expression of PELATON in PG-3 glioma primary cells after treatment with siRNAs (si226, si342, and si478), Datas were mean ± SEM for three independent experiments. (**p < 0.01). (F–G) RNA level changes of BACH1 and CD44 when PELATON was knocked down or overexpressed in glioblastoma cells, Datas were mean ± SEM for three independent experiments. (*p < 0.05, ***p < 0.001, ns: not significant). (H–I) Protein level changes of BACH1, CD44 and P53 when PELATON was knocked down or overexpressed in glioblastoma cells, independent experiment was repeated for three times. [file Image_3.tif]

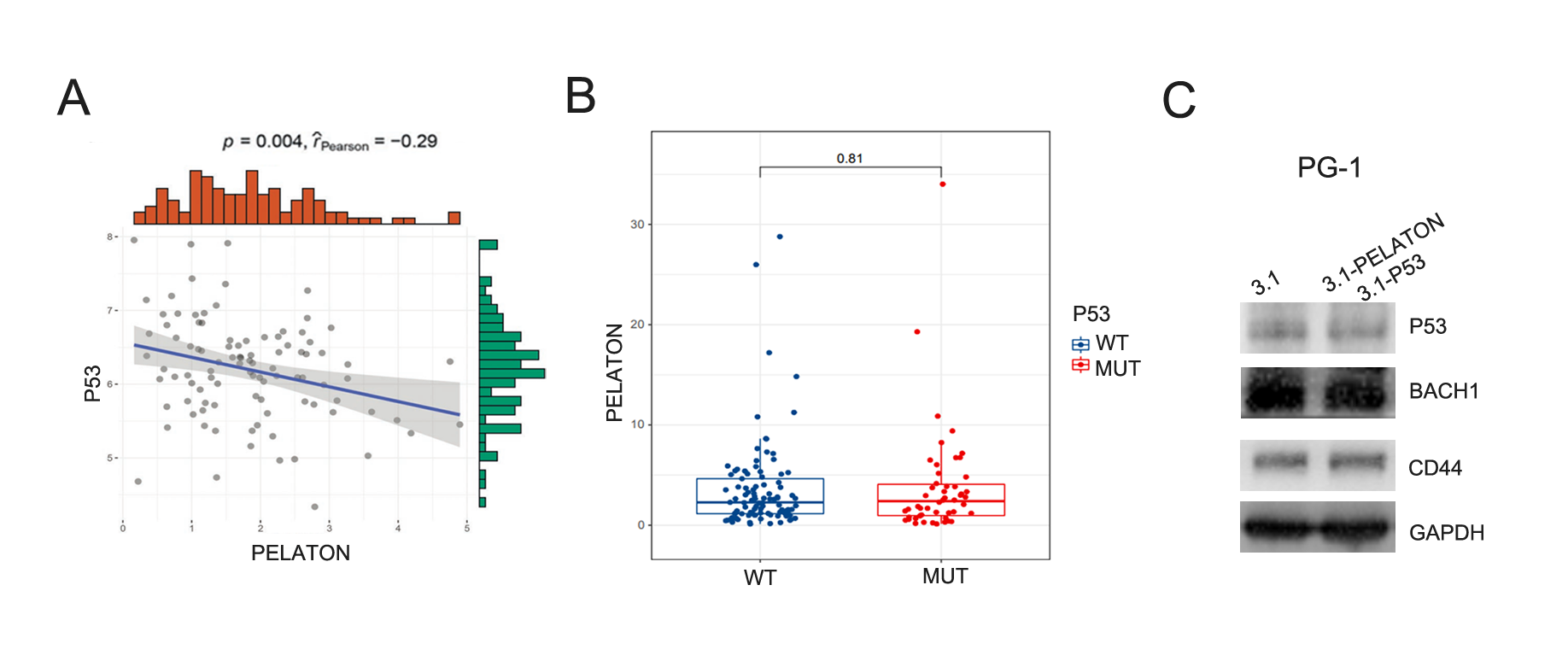

Supplement: Supplementary Figure 4 — Regulatory Relationship between PELATON and P53. (A) Bioinformatics correlation between PELATON and P53. (B) The difference in PELATON expression between wild-type and mutant P53 in GBM patients. (C) western blot analysis of BACH1, CD44 and P53 when PELATON and P53 were overexpressed in glioblastoma cells, independent experiment was repeated for three times. [file Image_4.tif]

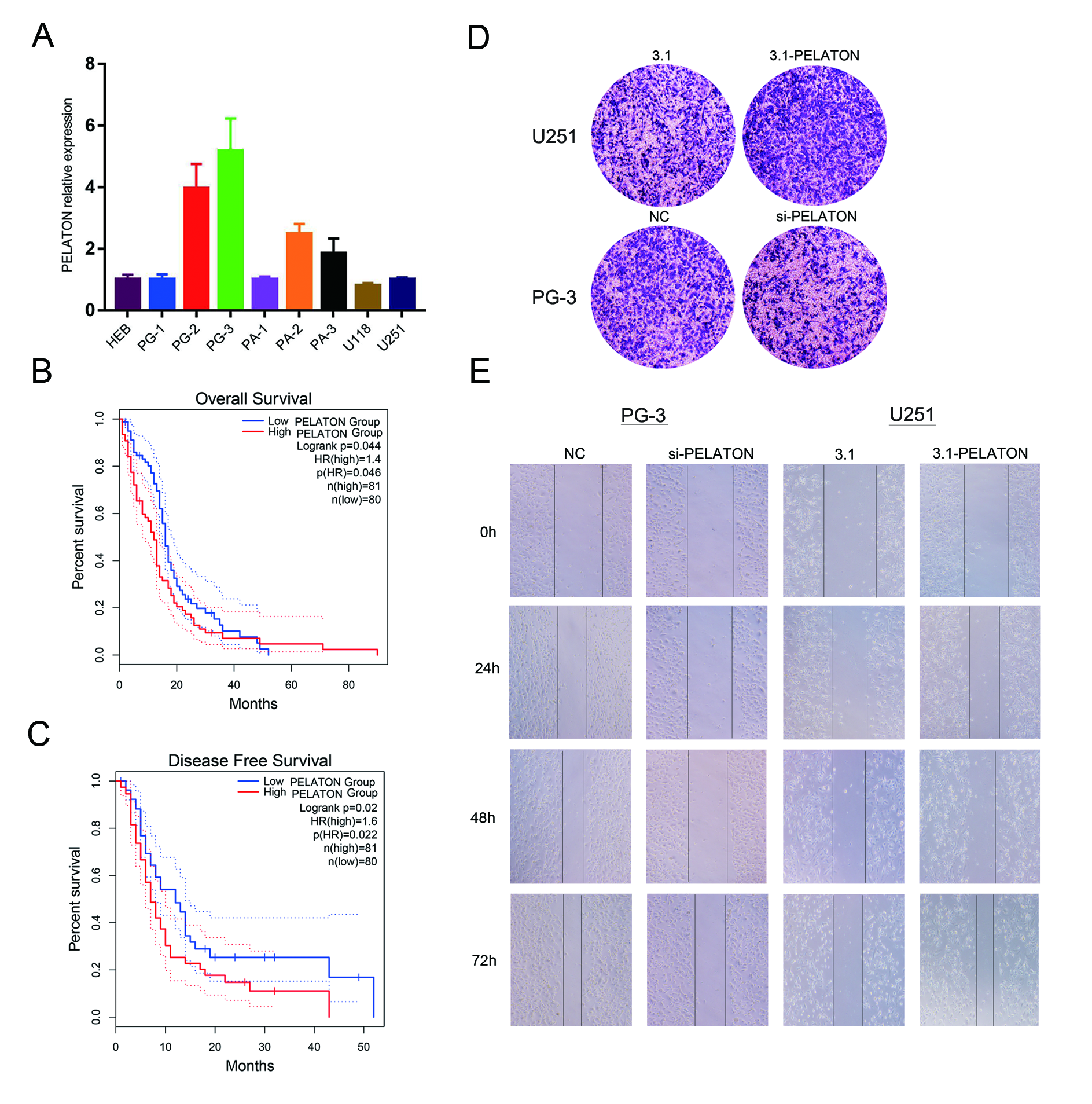

Supplement: Supplementary Figure 5 — Association of PELATON expression between patient prognosis and GBM cell phenotype. (A) RT-qPCR analysis of PELATON in glioma primary cells (PG-1, PG-2, PG-3, PA-1, PA-2, PA-3) and glioma cell lines (U118, U251). PG-1, PG-2, and PG-3 are primary cells from patients with glioblastoma, and PA-1, PA-2, and PA-3 are primary cells from patients with astrocytoma. Datas were mean ± SEM for three independent experiments. Kaplan–Meier curves showing overall survival (B) and disease-free survival (C) of patients with GBM stratified based on PELATON expression levels (p <0.05 p.adjust<0.25). (D) The effect of PELATON overexpression or knockdown on the invasion ability of glioblastoma cells (left), independent experiment was repeated for three times. (E) Migration ability of PG-3 and U251 cells after interference or overexpression with PELATON. Photos were taken at 0, 24, 48 and 72 hours, independent experiment was repeated for three times. [file Image_5.tif]
